# Uncertainty Estimation for Quantitative Agarose Gel Electrophoresis of Nucleic Acids

**DOI:** 10.3390/s23041999

**Published:** 2023-02-10

**Authors:** Konstantin Semenov, Aleksandr Taraskin, Alexandra Yurchenko, Irina Baranovskaya, Lada Purvinsh, Natalia Gyulikhandanova, Andrey Vasin

**Affiliations:** 1 Peter the Great St. Petersburg Polytechnic University, 29, Polytechnicheskaya Str., 195251 St. Petersburg, Russia; 2 Smorodintsev Research Institute of Influenza, 15/17, Prof. Popov Str., 197376 St. Petersburg, Russia; 3 Augusta University, 1120 15th Str., Augusta, GA 30912, USA; 4 The University of Chicago, 947 E. 58th Str., Chicago, IL 60637, USA

**Keywords:** quantitative gel electrophoresis, electrophoretic separation, nucleic acids, metrological analysis, uncertainty propagation, inaccurate data processing

## Abstract

This paper considers the evaluation of uncertainty of quantitative gel electrophoresis. To date, such uncertainty estimation presented in the literature are based on the multiple measurements performed for assessing the intra- and interlaboratory reproducibility using standard samples. This paper shows how to estimate the uncertainty in cases where we cannot study scattering components of the results. The first point is dedicated to a case where we have standard samples (the direct expressions are shown). The second point considers the situation when standard samples are absent (the algorithm for estimating the lower bound for uncertainty is discussed). The role of the data processing algorithm is demonstrated.

## 1. Introduction

Electrophoretic separation of nucleic acids in agarose gel [1,2] is a convenient way of obtaining quantitative information about the content of certain substances according to their size and structure in the analyzed sample [3,4]. Electrophoresis in agarose gel allows to separate DNA fragments with length from approximately 100 to 25,000 base pairs [5]. The paper [6] notes: “The wide range of applications makes agarose gel electrophoresis an extremely important technique. Although the recent advent of next-generation sequencing technologies has the potential to replace many of the current uses of agarose gels, their ease of use and versatility means that this technique is likely to persist for the foreseeable future”. Among the numerous applications of electrophoretic separation of nucleic acids in agarose gel, the following can be mentioned: studies on cancer [7,8], gene mutations [9,10], molecular alteration caused by radiation [11,12], etc. The wide use of the method is the reason for applying electrophoretic separation in agarose gel as a comparison standard when developing other quantitative analysis methods [13].

The use of this method to measure the amount of one or another of the mixture under study is associated with the distorting influence of a sufficiently large number of acting factors [14], which is typical for any measurement method used in biochemistry and biophysics. The paper [5] lists the following among the most important impacts on the rate of migration of nucleic acid molecules through agarose gel: the size of the nucleic acid molecule studied; agarose gel concentration; nucleic acid conformation; the voltage applied; the presence of ethidium bromide; the type of agarose gel; electrophoresis buffer. Consequently, to assess the total uncertainty of the final measurement results, it is necessary to conduct complex studies that would allow us to evaluate the contribution of each significant component to the resulting error. A statistically significant assessment of intra- and interlaboratory reproducibility requires a substantial number of measurements in the same and different conditions [15]. Estimating the errors associated with the preparation of specimens based on standard samples (with known composition and concentrations) according to standard protocols does not allow us to fully consider the contribution of the resulting methodological error (systematic bias in the results).

Studies performed to assess the utmost accuracy of measuring the content of various components in the test sample, as a rule, are carried out for a specific substance [16]. It is proven difficult to transfer the obtained estimates to a different (and even close) composition of the analyzed mixture, since the features of electrophoresis performance change significantly—starting from the sample preparation protocol and the dynamics of the electrophoretic separation process course and ending with the conditions for performing mathematical processing of electrophoresis images. This circumstance is substantial for the electrophoretic separation of nucleic acids, for which the studied samples are usually of a complex composition. For example, investigation of the in vitro transcription reaction under different conditions using electrophoretic separation is associated with even greater difficulties in assessing the uncertainty of the obtained results. In addition, as previously noted in Ref. [17], «the electrophoretic mobility of single-stranded DNA through agarose gels depends on size (number of bases; molecular weight), topology (linear or circular), and conformation (secondary and tertiary folding through base pairing and stacking), as well as on gel concentration and field strength».

In the existing literature, the issues of assessing the accuracy of the quantitative agarose gel electrophoresis of nucleic acids are insufficiently covered. The methods presented in published articles cannot be fully transferred to the above problem due to the impossibility of standardizing its conditions and the variability in the composition of nucleic acids. This does not allow the ultimate study of the accuracy of the results obtained using the traditional assessment of the individual components of intra- and interlaboratory reproducibility.

It is important to note that, in general, the question of studying the accuracy of the results of quantitative analysis using electrophoretic separation is usually limited to the study of reproducibility—and on a very small number of repetitions (usually no more than 30). The statistical uncertainty corresponding to the derived estimates of the standard deviation of the results’ random variation is usually not given, —although it is at least 25–30% for the noted conditions for a 95% confidence level. Thus, the practical use of various types of electrophoretic separations for quantitative analysis can often be associated with an overestimation of the marginal accuracy of the results achieved. A correct estimation of the uncertainty of the final results is the most important task in this field, without which it is impossible to perform assessments of the quality of the obtained results of quantitative measurements. At the same time, the state of works in electrophoretic separation is such that about 80% of them do not give any estimates of the error of the results; while the query “electrophoretic separation” + “agarose gel” in the scientific search system Google Scholar, whose syntax implies a mandatory mentioning of these phrases, returns 20,200 papers, the query “electrophoretic separation” + “agarose gel” + (“standard deviation” OR “standard error”) returns only 3960 papers (as of 2 February 2023). This seems to be quite a serious problem, since it constrains the possible applications and correct interpretations of the results obtained. To encourage researchers to provide appropriate estimates of uncertainty, the latter should be as easy as possible when processing the experiments performed. For this purpose, the corresponding estimate must either be expressed in simple relations that are easy to understand and use for calculations or in the form of software that automatically computes an uncertainty estimate for each result of the mathematical processing of the measurements performed. The present study takes a step in this direction and proposes appropriate approaches, as it seems to the authors convenient for practical application. Therefore, this article presents a method for estimating the uncertainty bounds of the quantitative results for the composition of substances during the electrophoretic separation of nucleic acids in agarose gel, which is appropriate for use when interlaboratory comparisons are impossible and when a small number of repeated measurements for assessing intralaboratory reproducibility are available.

## 2. Literature Review

The standard methods (common in analytical chemistry and biochemistry [15]) for estimating uncertainty use a statistical assessment of the multiple measurements’ variability. This approach involves construction of the so-called uncertainty profile based on confidence tolerance limits for a given content of the test substance (β-content, γ-confidence tolerance intervals). The boundaries of such intervals can be constructed approximately, based either on approximations [18,19], such as the Welch–Satterthwaite expression [20], or statistical modeling [21]. These methods are partially non-parametric, although they are based on some assumptions and premises, which are subject to reasonable criticism from the viewpoint of mathematical statistics [22,23,24,25]. At the same time, this approach is universal to a certain extent [26]—it can be used not only for the electrophoretic separation of nucleic acids but also for the results obtained by separation using chromatography and other analysis methods [15].

The approaches listed make it possible to build confidence intervals and to evaluate the quality of measurements performed using electrophoretic separation of nucleic acids. However, they require a significant number of repeated measurements to evaluate the reproducibility variance as the sum of intra- and interlaboratory frequency variance estimates. These methods evaluate only the uncertainty of the final result of a statistical nature associated with the influence of random factors and do not reflect the magnitude of the error corresponding with the method of mathematical processing used. Their application in practice allows one to obtain only a very approximate idea of the accuracy of the achieved results (in particular, the typical sample size for estimating statistical uncertainty is about 10 for interlaboratory comparisons [16]).

Some publications focus on studying the statistical uncertainty associated with a specific element of performing measurements related to electrophoretic separation—for example, determining the value of electrophoretic mobility (band position) [27]. Reproduction of the electrophoretic mobility scale during electrophoresis is carried out by introducing into one of the wells of the agarose gel an external standard containing known components with known mobility values. The correlation of the results of processing the standard with the results of processing the test sample allows one to obtain a mobility scale for the latter. In Ref. [28], it is noted that the level of the RMS error reaches values up to 0.2% (imaging component of measurement variability) and 0.4% (interlaboratory reproducibility) [27].

In the previously published paper [14], the following data are given on the recommended upper limits for the most important uncertainty components when validating the results of capillary electrophoresis on real samples: selectivity and specificity—(3–5)%; repeatability and intermediate precision—2.5%; total uncertainty factors from calibration—2%. At the same time, against these errors, Ref. [14] estimates the efficiency of nucleic acid separation (<15%) as well as the accuracy of peak identification and integration of their area (<10%) are relatively low. At the same time, the baseline noise level is estimated as capable of reaching sufficiently significant values too (<5%). It is important to note that these estimates indicate an error that arises due to the mathematical processing of the results of nucleic acids’ electrophoretic separation in order to determine their quantitative content. In current practice, this type of uncertainty is not taken into account at all during the calibration. This statement is true when the calibration and actual measurements are homogeneous, and also carried out under the same conditions, but given the features of the electrophoretic separation method, this is not always possible.

An important implication of these circumstances is that there is a significant resource for reducing errors due to the use of more advanced methods for the mathematical processing of electrophoresis images.

## 3. Materials and Methods

The methodological base of the presented study was to investigate the influence of the mathematical processing of electrophoresis images on the substance quantity measurements.

The processing of images acquired during nucleic acids’ electrophoretic separation in agarose gel is accompanied by a number of transformations and complex mathematical procedures, which result in a quantitative measurement of the content of different substances in the sample under study. The values obtained are subject to all the requirements for ensuring the traceability of measurements, prescribing to accompany the measurement result by an individual estimate of error or to indicate that the magnitude of the error did not exceed the limits set for it with a specified probability. The difficulty of full metrological support for measurements performed using electrophoretic separation of nucleic acids lies in the need to prepare sample mixtures with known component content; —this would make it possible to estimate with adequate accuracy the errors of the results obtained during electrophoresis image processing under real operating conditions of electrophoretic separation systems. Evaluation of in vitro transcription results with the use of electrophoretic separation is additionally associated with the emergence of reaction byproducts and non-target compounds with different electrophoretic mobility. Moreover, preparation of the corresponding reference samples is limited due to the difficulties of in vitro transcription reaction control. The absence or rarity of reference materials and reference devices in metrological practice is solved by estimating the uncertainty of measurement results through mathematical modeling and studying the error transformation through computational procedures.

The main components of uncertainty in the electrophoretic separation of nucleic acids in agarose gel are:

(1) instrumental errors Δ_1_ related to the physical realization of electrophoretic separation, quality of the agarose gel, the geometry of the well and during the placement of the sample (these sources of uncertainty cause both systematic bias in the measurement results and the variability of the results when performing multiple measurements);
(2) errors Δ_2_ associated with the mathematical processing *C* = *f*(*I*) of images obtained as a result of electrophoretic separation. These sources of uncertainty are associated with errors caused by determining the boundaries of bands corresponding to certain lanes, with the incorrect correlation of intensity distribution along the well with the electrophoretic mobility scale, with procedures of baseline removal and separation of overlapping peaks. Here, *C* is the result of the substance quantity measurement, and *I* is the processed image.

The reproduction of the scale of electrophoretic mobility in this case, due to the quantitative estimates presented in the previous paragraph of the article, can be removed from consideration of uncertainty sources due to the smallness of the introduced uncertainty against the background of its other components.

The upper bound for the total error of the final measurement result can be determined by the ratios

$$\left|\Delta \right|=\left|f\left(I+\Delta_{1}\right)-f\left(I\right)\right|+\Delta_{2}\;\;\left|\frac{df\left(I\right)}{dI}\right|\cdot \Delta_{1}+\Delta_{2}$$

Since the errors Δ_1_ are transformed when performing mathematical processing *f*, it is difficult to determine the distribution density for Δ in advance (since it is determined, among other things, by the sample under study; —the results of peak separation in the intensity distribution depend on the complexity of its composition). As a result, it is difficult to divide the error into systematic and random components, so that the limiting normalized values for them would be valid for any composition of nucleic acids subjected to separation.

If a sample provides the distribution *h*(*m*) with well-separated peaks as a result of electrophoretic separation, then it becomes possible to use the calibration procedure for the implementation of metrological support under some inconsequential assumptions. Here, *h* is the intensity plotted along the depth of the well with the substance under study, and *m* is the value of the electrophoretic mobility. If, however, the composition is sufficiently complex, calibration becomes difficult due to the lack of standard samples noted above. In this case, there is nothing left to do but to estimate the accuracy of the obtained electrophoretic separation results through calculation and mathematical modeling. Such a solution, in the absence of reference measures and means of calibration and verification, is not a distinctive feature of quantitative electrophoresis and is a common practice when there are no other ways to estimate the results accuracy. In this case, the result error estimation can be performed in two steps. First, the reproducibility of the intensity distribution in the well of interest (together with the magnitude of random variability) is evaluated by multiple repeats and comparing the images obtained in this case. Then, the data acquired are subjected to mathematical processing and evaluation of the transformation of the first stage result errors, taking place in the course of it. Additionally, errors arising during mathematical processing are evaluated with the help of mathematical modeling tools.

## 4. Results

### 4.1. Uncertainty Assessment in the Presence of Standard Samples of Separable Nucleic Acid Mixtures with Known Concentrations

If it is known from a priori information that the electrophoretic separation procedure is applied to a sample composed either of a single substance or of a mixture with a small number of well-separable components for which there are standard samples, then it is possible to use the whole electrophoretic separation system calibration procedure to assess the accuracy of the measurement results and to provide their metrological support.

The application of the following procedure is proposed for estimating the error in the results of quantitative measurements of the test sample content.

1. The electrophoretic separation system is calibrated for a sample of nucleic acids (provided that the electrophoretic mobility of the mixture components is different), whose concentration *C_i_*, *i* = 1, 2, …, *k* in the mixture is known with a relative error γ*_i_*. When calibrating the electrophoretic system for this sample, *n* repeated measurements of the corresponding peak areas in the signal *h*(*m*) are performed, the results of which are denoted as ${\hat{S}}_{ij}$, *j* = 1, 2, …, *n*. To eliminate random noise in the results of electrophoretic separation in the system used, a sensitivity threshold *C*_min_ is set, such that the contents of substances smaller than this threshold are not registered: if *C* < *C*_min_, the measurement result *S* = 0.

This circumstance leads to a negative systematic error in the calibration results of electrophoretic separation system, which is added to the systematic bias (if the latter takes place) and generates a total systematic error Δ*S*, possibly different for the different sample components studied.

As a result of the *i*-th calibration operation, we obtain

$${\hat{S}}_{ij}=K\cdot C_{i}-\Delta S_{i}+{\hat{\varepsilon }}_{ij}$$

where *K* is a coefficient of proportionality between the value of the peak area in the signal *h*(*m*) and the measured amount of substance content corresponding to it; ${\hat{\varepsilon }}_{ij}$ is a random error with zero mathematical expectation. The variability of values at the same *i* and different *j* is determined only by random reasons ${\hat{\varepsilon }}_{ij}$. The value of *K* should be evaluated in the course of calibration for the whole set of obtained measurement results using least squares techniques.

2. Immediately after calibration, the sample substance to be measured is introduced into the electrophoretic separation system. The sensitivity threshold set during calibration and, consequently, the absolute systematic error Δ*S* caused by this threshold, remain unchanged. Electrophoretic separation is performed several times to estimate random errors. It is reasonable to take the number of measurements equal to *n*—as being performed during the calibration. Accordingly, in the *i*-th analysis, the values of the area $S_{ij}$ will be obtained:
  $$S_{ij}=K\cdot C_{act\;i}-\Delta S_{i}+\varepsilon_{ij}$$

This expression is also perturbed only by random errors $\varepsilon_{ij}$ with zero mean. We assume that the systematic error Δ*S_i_* and the coefficient of proportionality *K* do not change during the calibration and measurement.

Random errors ${\hat{\varepsilon }}_{ij}$ and $\varepsilon_{ij}\;$ are independent of each other, so we conclude that there is no correlation between their values.

3. Next, the statistical processing of the results of multiple measurements when calibrating the electrophoretic separation system and when using it to measure the composition of the sample of interest is performed.

Accordingly, for each element of the studied sample, we calculate:

– mean values of the results of calibration and measurement
  $${\hat{M}}_{i}=\frac{1}{n}\cdot \sum_{j=1}^{n}{\hat{S}}_{ij},\;M_{i}=\frac{1}{n}\cdot \sum_{j=1}^{n}S_{ij},$$
– estimates of random error variances
  $${\hat{\sigma }}_{i}^{2}=\frac{1}{n-1}\cdot \sum_{j=1}^{n}{\left({\hat{S}}_{ij}-{\hat{M}}_{i}\right)}^{2},\;\sigma_{i}^{2}=\frac{1}{n-1}\cdot \sum_{j=1}^{n}{\left(S_{ij}-M_{i}\right)}^{2},$$
– estimates of the arithmetic means variances
  $${\hat{\sigma }}_{\mathrm{mean}\;i}^{2}=\frac{{\hat{\sigma }}_{i}^{2}}{n-1},\;\sigma_{\mathrm{mean}\;i}^{2}=\frac{\sigma_{i}^{2}}{n-1},$$
– upper bounds ${\hat{V}}_{\max i}$, $V_{\max i}$ of one-sided confidence intervals for variances of arithmetic mean with confidence probability *P* = 0.95 based on quantiles of $\chi^{2}$ distribution with the number of degrees of freedom equal to (*n* − 1)
  $${\hat{V}}_{\max\;i}=\frac{n-1}{\chi_{1-P}^{2}\left(n-1\right)}\cdot {\hat{\sigma }}_{\mathrm{mean}\;i}^{2},\;V_{\max i}=\frac{n-1}{\chi_{1-P}^{2}\left(n-1\right)}\cdot \sigma_{\mathrm{mean}\;i}^{2}$$
  (it is assumed that the electrophoretic mobility scale is aligned and represented on a linear scale),
– estimates of variances of relative random errors of mean values
  $${\hat{\gamma }}_{\mathrm{mean}\;i}^{2}=\frac{{\hat{V}}_{\max i}}{{\hat{M}}_{i}^{2}}=\frac{n-1}{\chi_{1-P}^{2}\left(n-1\right)}\cdot \frac{{\hat{\sigma }}_{\mathrm{mean}\;i}^{2}}{{\hat{M}}_{i}^{2}},\;\gamma_{\mathrm{mean}\;i}^{2}=\frac{V_{\max i}}{M_{i}^{2}}=\frac{n-1}{\chi_{1-P}^{2}\left(n-1\right)}\cdot \frac{\sigma_{\mathrm{mean}\;i}^{2}}{{\hat{M}}_{i}^{2}}.$$

4. The content $C_{acti}$ of the *i*-th component of the test sample is calculated as the solution to the system of equations from steps 1 and 2 of this list:
  $$\{\begin{array}{c} {\hat{M}}_{i}=K\cdot C_{i}-\Delta S_{i},\\ M_{i}=K\cdot C_{act\;i}-\Delta S_{i}. \end{array}$$
  Here, we have two unknowns ($C_{act\;i}$, $\Delta S_{i}$) and two equations, so this system is well conditioned. Therefore,
  $$C_{act\;i}=\frac{M_{i}+\Delta S_{i}}{{\hat{M}}_{i}+\Delta S_{i}}\cdot C_{i}\;\mathrm{or}\;C_{act\;i}=C_{i}+\frac{M_{i}-{\hat{M}}_{i}}{K}.$$
5. The expression for the relative error (uncertainty) of the results of measuring the quantity of a substance when using electrophoretic separation is the sum
  (1) $$\frac{\Delta C_{act\;i}}{C_{act\;i}}=\frac{\Delta {\hat{M}}_{i}}{{\hat{M}}_{i}+\Delta S_{i}}+\frac{\Delta M_{i}}{M_{i}+\Delta S_{i}}+\frac{\Delta C_{i}}{C_{i}}\approx \frac{\Delta {\hat{M}}_{i}}{{\hat{M}}_{i}}+\frac{\Delta M_{i}}{M_{i}}+\frac{\Delta C_{i}}{C_{i}}$$
  under assumptions that $\left|\Delta S_{i}\right|\ll M_{i}$ and $\left|\Delta S_{i}\right|\ll {\hat{M}}_{i}$, or
  $$\Delta C_{act\;i}=\Delta C_{i}+\frac{\Delta M_{i}-\Delta {\hat{M}}_{i}}{K}-\frac{\left(M_{i}-{\hat{M}}_{i}\right)\cdot \Delta K}{K^{2}},$$
  (2) $$\frac{\Delta C_{act\;i}}{C_{act\;i}}=\frac{\Delta C_{i}}{\frac{M_{i}+\Delta S_{i}}{{\hat{M}}_{i}+\Delta S_{i}}\cdot C_{i}}+\frac{\Delta M_{i}-\Delta {\hat{M}}_{i}}{\frac{M_{i}+\Delta S_{i}}{{\hat{M}}_{i}+\Delta S_{i}}\cdot C_{i}}-\frac{\left(M_{i}-{\hat{M}}_{i}\right)\cdot \Delta K}{\frac{M_{i}+\Delta S_{i}}{{\hat{M}}_{i}+\Delta S_{i}}\cdot C_{i}\cdot K^{2}}\approx \frac{\Delta C_{i}}{C_{i}}\cdot \frac{M_{i}+\Delta S_{i}}{{\hat{M}}_{i}+\Delta S_{i}}+\frac{1}{C_{i}}\cdot \left({\hat{M}}_{i}\cdot \frac{\Delta M_{i}}{M_{i}}-\frac{{\hat{M}}_{i}^{2}}{M_{i}}\cdot \frac{\Delta {\hat{M}}_{i}}{{\hat{M}}_{i}}-\left(M_{i}-{\hat{M}}_{i}\right)\cdot \frac{{\hat{M}}_{i}+\Delta S_{i}}{M_{i}+\Delta S_{i}}\cdot \frac{\Delta K}{K}\right)$$

The first two terms in Equation (1) reflect the independent random errors and form the uncertainty estimated by type A. The third term is a systematic error; —its value is in the interval normalized for the sample mixture. This summand forms the uncertainty processed by type B.

The expression (2) contains the estimate of the relative error of proportionality coefficient *K*, which depends on data from the calibration and measurements, and this is why it is correlated with errors of ${\hat{M}}_{i}$. This circumstance should be considered during application (2) to prevent the underestimation of uncertainty bounds.

As a consequence of Equation (1), the final estimate of the marginal relative error of the results of substance quantity measurement by electrophoretic separation of nucleic acids in agarose gel using calibration in the presence of standard samples for a confidence probability of 0.95 satisfies the following inequality.

(3) $$\left|\frac{\Delta C_{act\;i}}{C_{act\;i}}\right|\leq g\cdot \sqrt{{\hat{\gamma }}_{\mathrm{mean}\;i}^{2}+\gamma_{\mathrm{mean}\;i}^{2}}+\left|\frac{\Delta C_{i}}{C_{i}}\right|\leq 2\cdot \sqrt{\frac{n-1}{\chi_{1-P}^{2}\left(n-1\right)}}\cdot \sqrt{\frac{{\hat{\sigma }}_{\mathrm{mean}\;i}^{2}}{{\hat{M}}_{i}^{2}}+\frac{\sigma_{\mathrm{mean}\;i}^{2}}{{\hat{M}}_{i}^{2}}}+\gamma_{i}$$

The value of *g* is a coefficient of uncertainty coverage and, according to Refs. [29,30], should be taken as two for a wide family of distributions that differ little from the normal one, in case the confidence probability is estimated to be 0.90 ÷ 0.95.

The expression (2) brings us to the following estimate for the uncertainty bound:

(4) $$\left|\frac{\Delta C_{act\;i}}{C_{act\;i}}\right|\leq \left|\frac{\Delta C_{i}}{C_{i}}\right|\cdot \frac{M_{i}+\Delta S_{i}}{{\hat{M}}_{i}+\Delta S_{i}}+\frac{1}{C_{i}}\cdot \left(g\cdot \sqrt{{\hat{M}}_{i}^{2}\cdot {\hat{\gamma }}_{\mathrm{mean}\;i}^{2}+\frac{{\hat{M}}_{i}^{4}}{M_{i}^{2}}\cdot \gamma_{\mathrm{mean}\;i}^{2}}+\left|M_{i}-{\hat{M}}_{i}\right|\cdot \frac{{\hat{M}}_{i}+\Delta S_{i}}{M_{i}+\Delta S_{i}}\cdot \frac{\Delta K}{K}\right)\leq \;\leq \left|\frac{\Delta C_{i}}{C_{i}}\right|\cdot \frac{M_{i}+\Delta S_{i}}{{\hat{M}}_{i}+\Delta S_{i}}+\frac{1}{C_{i}}\cdot \left(2\cdot \sqrt{\frac{n-1}{\chi_{1-P}^{2}\left(n-1\right)}}\cdot \sqrt{{\hat{\sigma }}_{\mathrm{mean}\;i}^{2}+\frac{{\hat{M}}_{i}^{2}}{M_{i}^{2}}\cdot \sigma_{\mathrm{mean}\;i}^{2}}+\left|M_{i}-{\hat{M}}_{i}\right|\cdot \frac{{\hat{M}}_{i}+\Delta S_{i}}{M_{i}+\Delta S_{i}}\cdot \delta \right),$$

where δ is the maximum possible value of the relative uncertainty of the sensitivity coefficient *K*. Its value depends on the way the estimate for *K* is performed.

The expressions (3) and (4) obtained estimate the total error of the quantitative result of measuring the substance’s content in the sample under study using electrophoretic separation of nucleic acids in agarose gel.

### 4.2. Uncertainty Assessment for a Case Where There Are No Standard Samplesthe Case When There Are No Standard Samples of the Nucleic Acid Mixture to Be Separated with Known Concentrations

The previous case considered generally corresponds to a situation where, according to the results of image processing obtained during electrophoretic separation of nucleic acids in agarose gel, the signal *h*(*m*) represents a distribution with well-separated peaks, i.e., such that between two neighboring peaks, there is a gap of non-zero width, for each point *m* of which *h*(*m*) = 0.

Now, suppose that the signal *h*(*m*) contains intersecting peaks, i.e., there is at least one interval $\left[m_{1},m_{2}\right]$, such that it includes more than one point where the derivative of the signal *h*(*m*) is zero, but the signal *h*(*m*) itself is nevertheless non-zero.

The value of the measurement result of the amount of substance’s content in the test sample is determined by applying the mathematical processing procedure *C* = *f*(*I*), which performs the following steps:

– processing of the image of intensity distribution along the depth of the well in the agarose gel obtained during electrophoretic separation (alignment, rotation, removal of local geometric deformations, compensation of the background intensity in the processed image fragment);
– a statistical integrated estimate of the intensity within each line of the well’s image and formation of signal *h*(*m*), where *m* is the value of the electrophoretic mobility corresponding to each line of the image;
– removal of the underlying background and baseline *h*_0_(*m*) using the construction of the lower envelope for the signal *h*(*m*);
– applying of the iterative procedure for separating the overlapping peaks in the signal (*h*(*m*) − *h*_0_(*m*)).

To assess the instrumental errors associated with the physical implementation of electrophoretic separation, the quality of the agarose gel plate, the well’s geometry and sample placement, multiple repetitions of electrophoretic separation are performed for several identical samples of the nucleic acid mixture under study.

The resulting images *I*_1_, *I*_2_, … *I_n_* are compared with each other as follows. Their average *I*_mean_, obtained by pointwise averaging the obtained images’ sample, is considered. The alignment of the two images is performed by finding such horizontal and vertical (if required) offset, which provides the maximum of the mutual correlation function of the compared images:

$${\tilde{I}}_{i}\left(x,y\right)=I_{i}\left(x-d_{x},y-d_{y}\right),$$

$$\left(d_{x},d_{y}\right)=\arg\underset{r_{x},r_{y}}{\max}\sum_{x}\sum_{y}\bar{I}\left(x,y\right)\cdot I_{i}\left(x-r_{x},y-r_{y}\right),$$

where *x* and *y* are the row and column indices, respectively, $\bar{I}=\frac{1}{n}\cdot \sum_{i=1}^{n}I_{i}$.

Then,

$$I_{\mathrm{mean}}=\frac{1}{n}\cdot \sum_{i=1}^{n}{\tilde{I}}_{i}.$$

For each point of the images combined in this way, the variance of the random scatter is estimated:

$$\sigma^{2}\left(x,y\right)=\frac{1}{n-1}\cdot \sum_{i=1}^{n}{\left({\tilde{I}}_{i}\left(x,y\right)-\bar{I}\left(x,y\right)\right)}^{2}.$$

Accordingly, these values can be used as estimates of the random error for each point of the averaged images:

$$\sigma_{\mathrm{mean}}^{2}\left(x,y\right)=\frac{\sigma^{2}\left(x,y\right)}{n}.$$

These estimates are used for further transformations as follows. Let *C* be the final result of measurements, i.e., an estimate of the concentration value of the component of interest in the sample under study. Then, the estimate of the limit of the random error of value *S*, inherited by it from the processed images’ errors, is equal to

(5) $$\left|\Delta_{\mathrm{rand}\;}C\right|\leq g\cdot \sqrt{\sum_{x}\sum_{y}{\left(\frac{\partial f\left(I_{\mathrm{mean}\;}\right)}{\partial I_{\mathrm{mean}\;}\left(x,y\right)}\right)}^{2}\cdot \sigma_{\mathrm{mean}\;}^{2}\left(x,y\right)},$$

under the assumption of independence of the results of the repeated electrophoresis performed. Here, the coverage factor *g* equals two on the grounds given above.

Executing the procedure *f* for the initial images *I*_1_, *I*_2_, … *I_n_* and estimating the error value *S* for a sample of the obtained results is a less good option because the algorithm *f* is significantly sensitive to the error value of the initial data (it contains procedures for solving incorrect problems). Their significant values can lead to distortions in assessing the underlying background and baseline, failure to detect hidden peaks, and so on. Since $\sigma_{\mathrm{mean}}^{2}$ is significantly less than the value of $\sigma^{2}$ at each point (*x*, *y*), the processing of image $I_{\mathrm{mean}}$ will be free from these drawbacks and, in addition, will require less time than the processing of all images *I*_1_, *I*_2_, … *I_n_*. Such circumstances lead to the necessity of processing a censored sample, for which the above-mentioned rule, such as Equation (5), is not always valid.

Further processing of the resulting image $I_{\mathrm{mean}}$ requires removing the baseline. If the researcher has a priori information regarding the range of electrophoretic mobility values corresponding to the substances present in the treated sample, the residual background intensity will be an estimate of the systematic error in the values (*h*(*m*) − *h*_0_(*m*)). Since the processed spectrogram must contain strictly positive values, in practice, the underlying background is estimated by plotting the lower envelope of the signal *h*(*m*). The values (*h*(*m*) − *h*_0_(*m*)) at *m*, corresponding to the forbidden values of electrophoretic mobility, estimate the limit of possible systematic error $\Delta_{\mathrm{syst}}$(*m*).

Let us denote the further signal transformation procedure (*h*(*m*) − *h*_0_(*m*)) as *f_h_*. Its result is the estimated content *C* of substance

$$C=f_{h}\left(h\left(m\right)-h_{0}\left(m\right)\right).$$

The limit of systematic error of the value *C* can be estimated similarly to the above, as follows:

(6) $$\left|\Delta_{\mathrm{syst}}C\right|\leq {\bar{\Delta }}_{\mathrm{syst}}\cdot D+\sum_{m}\left|\frac{\partial C}{\partial f_{h}\left(h\left(m\right)-h_{0}\left(m\right)\right)}\right|\cdot \left|\Delta_{\mathrm{syst}}\left(m\right)-{\bar{\Delta }}_{\mathrm{syst}}\right|,$$

where ${\bar{\Delta }}_{\mathrm{syst}}$ is the average value of the systematic error limit, and *D* is equal to the length of the interval of electrophoretic mobility values corresponding to the carrier of the peak in the spectrogram *h*(*m*) describing the analyzed component of the sample.

To compute the estimates (5) and (6) within a linear approximation, it is necessary to calculate the values of the derivatives. Standard techniques, such as the finite difference method, lead to poorly controlled computational errors and strongly depend on perturbations of the processed data, which, as noted above, are undesirable. For this reason, we should use the complex-step method [31], which allows us to obtain estimates of derivatives for an arbitrarily complex computational algorithm with accuracy up to rounding errors when representing floating-point numbers. The details of this approach are outlined in Appendix A. To apply this approach, it is necessary to ensure that all calculations performed in function *f* are performed without using complex numbers.

The mathematical formulation of the peak separation problem can be briefly summarized as follows. Any spectrogram *h*(*m*) recorded using the electrophoretic separation of nucleic acids in agarose gel is a convolution of the actual intensity distribution along the electrophoretic mobility values obtained in the analysis of a complex substance (or, for short, its spectrum *h_act_*(*m*)) and the impulse response function *r*(*m*) of the electrophoretic nucleic acid agarose gel separation system:

$$h\left(m\right)=\int_{0}^{+\infty }r\left(m-u\right)\cdot h_{act}\left(u\right)\;du.$$

As a result, the peaks registered in the spectrogram always widen and, in some cases, overlap, which makes it difficult to determine the area of each peak separately, although this parameter is the target for measurements and allows estimating the quantitative content of the analyzed substance during transcription. The solution of the presented equation for the target spectrogram *h_act_*(*m*) is an ill-conditioned problem, known in mathematics as the problem of solving the Fredholm integral equation of the first kind.

The use of Fourier-type transformations brings this equation to the form *H*(*j*ω) = *R*(*j*ω)·*H_act_*(*j*ω), where *j* is an imaginary unit, and ωω is a circular frequency. To avoid complex numbers, one must use one of the following techniques:

– using symmetrization of the transformed signals by reflecting them from the semiaxis of real values *m* > 0 to the semiaxis of negative values of the argument *m*, i.e., *h*(–*m*) = *h*(*m*) and *r*(–*m*) = *r*(*m*), and using the cosine transform, leading to strictly real-value spectra, instead of the integral Fourier transform;
– using the Hartley integral transform, —akin to the Fourier and the cosine transform.

In any of these cases, the final equation to be solved in the frequency domain will no longer be related to complex numbers and will be of the form *H*(ω) = *R*(ω)·*H_act_*(ω).

Its solution with respect to *h_act_*(*m*) should result in narrowing of the peaks and their separation, which is necessary to isolate the main peak responsible for the target yield of the transcription products and the side peaks responsible for the yield of side products on the spectrogram obtained from the electrophoretic separation instrument for nucleic acids in agarose gel. This problem is incorrect. The proposed solution uses the Tikhonov regularization method [32] reformulated as the minimal modulus principle [33], which is conveniently implemented in the domain of Fourier, cosine or Hartley transforms. However, its exact implementation leads to the Gibbs effect, which consists of the emergence of solution distortions in the form of oscillatory components in the recovered signal. To avoid this, we should apply a modification of the Howard algorithm [34], which leads to minimization of the negative values of *h_act_*(*m*). This procedure is an iterative process, in its original formulation, based on the use of forward and inverse Fourier transforms. In our case, one of the points of modification of the Howard algorithm is the use of transforms equivalent to Fourier transforms, which provide real-valued results (the already mentioned symmetrization and cosine transform or the Hartley transform). The procedures listed, which are the essence of algorithm *f*, keep the peak areas unchanged, which makes it possible to ensure the reliability of the results of measuring the concentration of substances in the sample analyzed using the electrophoretic separation of nucleic acids in agarose gel. In the presented formulation, however, no transformations involving an analytic continuation to the complex plane are used, which leaves the possibility of accompanying the calculation of f values with the automatic obtaining of values of partial derivatives of a given function and thereby automating the obtaining of estimates using expressions (5) and (6).

Since the boundaries of the possible systematic error involved in formula (5) may be significant, to ensure that the uncertainty of *C* is not excessively overestimated or underestimated due to the lack of consideration of derivatives of orders greater than one, it is possible to make an estimation using the Monte Carlo method. This approach involves a large amount of computing but does not require the errors transformed by the analyzed computational procedure to be small. The variant of the Monte Carlo technique recommended for use in metrological applications in the guide [35] is not the best option for the case described in the paper presented. The modification of this approach suitable for the electrophoresis image processing problems is presented in Appendix B.

The uncertainty estimates obtained in this way allow us to conclude to what extent the obtained quantitative values of nucleic acid content in the analyzed sample are perturbed by errors. Since the proposed approach is based entirely on the study of the transformation of errors present in the electrophoresis images, its results substantially reflect the uncertainty of imaging measurements. The main advantage of the proposed approach is that it can be used for single electrophoresis. Its application requires reasonable methods for processing the resulting intensity distributions, allowing both to remove the baseline and to reliably separate overlapping and detect hidden peaks.

## 5. Discussion

The issue of determining the accuracy of quantitative analysis using electrophoretic separation of nucleic acids in agarose gel and related methods has been studied rather fragmentarily in the literature—and was usually limited to the reproducibility of results. Such situation is partly due to the large number of factors influencing the final accuracy of electrophoretic separation results and partly due to the difficulty of conducting the corresponding experiments. However, even when only reproducibility is studied, the usual number of statistical repeats (5 ÷ 30) produced to estimate the inaccuracy of quantitative analysis based on electrophoretic separation should be recognized as insufficient for presenting the results without estimating the corresponding statistical uncertainty, which is substantial. Many practical situations allow performing only a few repeated measurements, which close the possibility of constructing uncertainty profiles. These circumstances indicate the need for new approaches and research in the metrological support of electrophoretic separation results.

In this paper, we present ratios that make it possible to standardize the accuracy limits of the results obtained for quantitative analysis of nucleic acids using electrophoretic separation in agarose gel, which take into account, among other things, the statistical error.

In [36], the following was noted: “Using internal standards can also help to improve peak area precision. In many cases the relative standard deviation (RSD) has been reported at <1%, for peak areas. Many manufacturers quote around 2% RSD for corrected areas, but with an experienced operator this can be improved to below 1%”. These estimates are in good agreement with the results of other works [37,38,39,40], but, at the same time, they do not contain requirements or indications regarding the number of repeated measurements necessary to achieve this accuracy. The mechanical transfer of these estimates to a smaller measurement number will lead to an underestimation of the final uncertainty of the results, which is unacceptable because it misleads the user about the measurement results. Since, in practice, it may not be possible to perform a sufficiently large number of repeated measurements, the ratios for error estimation must include the number of measurements performed as a parameter. The relations (3) and (4) presented in the article satisfy this requirement. Moreover, in some cases, they allow us to solve the inverse problem: to estimate a sufficient number of measurements to achieve the required accuracy, taking into account the influence of other factors and sources of error.

The case of standard samples’ absence leads to difficulties when using relations (3) and (4) because not all values in them appear to be known. In such a case, if the corresponding measurements were conducted by an experienced researcher familiar with the experimental equipment and past results obtained in the same laboratory under similar conditions, one may cautiously suggest using the expert estimates for the missing error components. In fact, a similar indication is implied in the quote from Ref. [36] above in this section. Formulae (3) and (4) can be easily generalized in this case to use fuzzy variables [41], the Dempster–Shafer theory [42] or other formalisms of expert information representation [43]. This will allow keeping the information about the objective part of the measurement results’ uncertainty estimate for a user and giving information about the subjective component. It seems that such a variant of uncertainty estimation is better than its complete absence (which, nowadays, is characteristic for most works in the field).

The method proposed in the Section 4.2 for estimating the uncertainty in the results of peak area estimation of the curves obtained during electrophoretic separation in agarose gel is the only possible approach when neither repeated measurements nor the use of standard samples are possible. This approach allows estimating the uncertainty of peak area estimation, which is inherited by it from all previous stages of the study and shows how, first of all, the instrumental errors of the technical means used turn into the error of final results of measurements. Information concerning the instrumental errors’ margins should be taken from the technical documentation of the instruments used. In fact, this approach implements the “uncertainty propagation” approach, well known and proven in metrology for performing calculations with inaccurate data. The obtained error estimates are estimates from below because they may not consider those errors that arise as a result of the not quite proper or ideal application of the technical means. However, the proposed procedure opens up the possibility of estimates based on mathematical modeling—a novel way not used before in electrophoretic separation metrology.

Paper [36] notes the following causes of incorrect estimation of the concentration of the substances under study when calculating the peak areas for the curves obtained by capillary electrophoretic separation: temperature changes, sample matrix evaporation, sample carry-over, zero injection caused by dipping capillary in sample solution, low signal to noise ratio, sudden application of high voltage, concentration-dependent peak shapes, intercapillary peak area changes, hydrodynamic injection, electrokinetic injection. Some of these reasons are also typical for separation in agarose gel. If these effects are controlled in the laboratory, it is possible to consider them when using the approach proposed in the paper presented. It is even possible to estimate the sensitivity of the accuracy of the final result to the corresponding error components.

The paper [36] notes that the relative standard deviation in measurements of small nucleic acid concentrations (0.01–2 mg/mL) is usually at least 2%. This work also states that the precision can be optimized using appropriate procedures to give a relative standard deviation of less than 1%. Refs. [44,45,46] contain a slightly more pessimistic estimate of the achievable accuracy in analyzing the diagnostic accuracy of electrophoresis. The error estimates quoted refer primarily to the procedure for calculating the peak area, since it mainly determines the accuracy of a low-concentration measurement result. The developed makes it possible to accompany the estimation of small concentration with an individual evaluation of its accuracy, which, among other things, will make it possible for a diagnostician to understand whether there are grounds to believe that the substance of interest is really in the sample under study or whether the detected non-zero concentration is just a product of a freakish concurrence of random errors.

The presented for estimating the uncertainty in the results of gel electrophoresis image processing allows one to look at previously published data from a new perspective. In particular, the fundamental limit to the accuracy of chemical analysis noted in Ref. [47] in the example of electrophoretic single molecules detection acts as a natural limit to the uncertainty of the substance content measurement from below. The approach presented in the article makes it possible to obtain a constraint from below for the applied methods of mathematical processing of electrophoresis images, which could provide the lowest uncertainty of the results of quantitative measurement of content and present it in the units of the mentioned resolution limit.

The uncertainty estimate Δ*C* presented in the paper reflects the error inherited from inaccuracies in the images obtained by electrophoresis. Consequently, its value shows the transformation of these errors during the mathematical processing of this image. There appears a possibility for objective choice of separate elements of the algorithm used; based on a comparison of the results of different methods of noise filtering, baseline elimination and peak separation, it is possible to choose an algorithm, which provides the lowest error inherited from the uncertainty of initial processed data and the best robustness of results, understood as the lowest sensitivity to the growth of these errors.

The main limitations of the study performed are the following:

– the need to involve information about the accuracy of standard samples (if used);
– the need to perform multiple measurements to achieve better accuracy (if they are possible);
– the need for analysis of technical documentation to identify all available information on all components of instrument uncertainty for the instruments used;
– the need to clearly distinguish between random and systematic components of the uncertainty budget for quantitative results derived from electrophoretic separation of nucleic acids in agarose gel;
– when applying the approach based on numerical modeling of peak area estimation, a significant number of calculations are required (this can pose a challenge when automating electrophoresis).

The practical application significance of the proposed approaches lies in the presentation of standardized ratios, which can be uniformly used to obtain uncertainty estimates for the quantitative results of electrophoretic separation of nucleic acids; —this opens the door to further unification and standardization in electrophoresis applications. The presented approach to the accompaniment of algorithms for processing images obtained during electrophoresis makes it possible, for the first time, to accompany this procedure with an estimate of uncertainty propagation and to provide, together with the concentration measurement result, an assessment of its uncertainty inherited from the technical means used for electrophoresis.

## 6. Conclusions

This paper considers the evaluation of uncertainty in the results of quantitative electrophoretic separation of nucleic acids in agarose gel. The uncertainty estimates of such measurements presented in the literature are based on multiple measurements using standard samples to assess the inter- and intralaboratory reproducibility of results. This article presents a new approach for estimating this uncertainty in cases where there are insufficient grounds to correlate the measurement results of a complex mixture of nucleic acids with the results of a pre-calibration performed on a standard sample and where no standard samples exist in principle. In the latter case, the need to fully consider the mathematical processing algorithm used for electrophoresis images is demonstrated.

## Data Availability

Not applicable.

## Appendix A. The Complex-Step Method for Derivatives Estimation

The complex-step method is a variant of the automatic differentiation of functions to be computed. Its implementation does not require specially introduced data types or substantial software development. Let us explain the idea behind this method using the example of some function *q*(**x**) from multiple real-valued arguments $x^{T}=\left(x_{1},x_{2},\ldots ,x_{k}\right)$. If this function permits the analytical continuation to the complex plane for variable *x_t_*, then we can use the complex-step approximation $est_{C}$ of partial derivative $\partial q\left(x\right)/\partial x_{t}$, which is much more accurate than finite difference estimate $est_{R}=\frac{q\left(x+d_{t}\right)-q\left(x\right)}{\alpha }$ and can be calculated as estC=Im(q(x+j⋅dt))α. Here, **d*_t_*** is a vector with the same size with **x** filled with zeros, except for its t-the element equal to some small number α~0, and $j=\sqrt{-1}$ is an imaginary unit.

In computing $est_{R}$, we usually take step α as the relative part of *x_t_* (α = 10^−*w*^·*x_t_*, where *w* is usually equal to 10 ÷ 20) to prevent a situation where the derivative estimate will be equal to zero due to the difference (*q*(**x** + **d*_t_***)–*q*(**x**)) being less than the machine precision allows to represent. This can lead to a significant final error. On the contrary, in computing $est_{C}$, we can always take α equal to 10^−200^, and such decision will not cause errors in derivative estimation. The main reason is that we are not faced, in that case, with computationally unstable operations, such as the subtraction of two close numbers, as it takes place in finite difference calculation.

The structure of the expression for $est_{C}$ is the same as for finite difference but is free from its disadvantages. The main idea is to estimate the derivative value using the imaginary step instead of the real-value step.

$$est_{C}=\frac{q\left(x+j\cdot d_{t}\right)-q\left(x\right)}{j\cdot \alpha }\approx \frac{\partial q\left(x\right)}{\partial x_{t}}.$$

Since the value of the derivative $\partial q\left(x\right)/\partial x_{t}$ must be a real number, the estimate can be transformed into the form

estC=Re(estC)=Im(q(x+j⋅dt)−q(x))α,

where Re(*z*) and Im(*z*) are a real and an imaginary component of the complex number *z*.

We know that the number *q*(**x**) is real, so we end up with the following expression:

estC=Im(q(x+j⋅dt))α.

The advantage of the complex-step approach is that the modern software packages for data processing permit the analytical continuation of all functions that occur automatically when its argument becomes a complex number. For example, in Matlab, we should only use commands such as “exp(x + 1i*y)” instead of “exp(x)” to transit from the real argument *x* to complex *z* = *x* + *j*·*y*.

Let us apply the complex-step method to expression (3) from the main part of the paper. The analytical continuation of the function *f* is carried out from the set *R^a^^×b^*, where *a* and *b* are the dimensions of the processed image *I*, to the set *C^a^^×b^* of complex numbers. Since the function *f* reflects a computational algorithm, this continuation is possible. Therefore,

∂f(I)∂I(x,y)≈Im(f(I+j⋅α⋅E(x,y)))α.

where *E*_(*x*,*y*)_ is a matrix of the same dimensions as image *I*, composed of zeros, except for the cell with indices (*x*, *y*), which contains one.

This expression allows us to estimate the values of all first-order partial derivatives with an error at the level of rounding errors.

## Appendix B. The Monte Carlo Method for Estimating Uncertainty Bounds

The Monte Carlo technique is widely used for propagating uncertainty in computations. The most popular variant of this method is based on studying the transformation of probability distributions from the uncertain initial variables to the results of calculations using random number generators. Such an approach supposes that distributions for the sources of uncertainty are known. If not, we are forced to make different assumptions with varying degrees of validity. This reduces confidence in the result.

The Monte Carlo method can be used not for estimating the distribution in full but for assessing the bounds of its support, —i.e., the interval of random variable possible values. In this case, the only necessary information is the limits of input data values caused by the uncertainty. This variant of the Monte Carlo approach is fully non-parametric and does not require any assumptions on probability distributions. The number of Monte Carlo iterations can be effectively reduced in this case by comparison with commonly given recommendations [35].

Estimating the support of distribution is equivalent to finding the minimum and maximum values of the function. Therefore, the Monte Carlo approach in this case is equivalent to the stochastic way of solving the optimization problem.

Let us consider the function *f* from the main part of the article. Let us find the minimum and maximum values of *f* for the area Ω of possible values of its arguments, determined by their errors: $J=\left[\min_{I\in \Omega }f\left(I\right),\;\max_{I\in \Omega }f\left(I\right)\right]$.

The essence of the Monte Carlo method is to use random covering for Ω. The main idea of the approach is that with random generation of *N* combinations of argument values, the probability that with *N*→∞ among them there will be those at which the value of function *f* will be close to the minimum or maximum value on Ω is close to 100%.

The algorithm of the Monte Carlo method boils down to the following sequence of operations.

1. To calculate the value of the function *C*_0_ = *f*(*I*) corresponding to the initial set *I* of argument values for *f*.
2. To generate *N* random combinations *I_j_* of argument values of the calculated function *f*; index *j* runs the values 1, 2,…, *N*; the generation is conducted according to a uniform distribution, since, in the general case, there is no reason to prefer some values from Ω over Ω to others in the absence of information about the function *f*.
3. To compute values *C_j_* = *f*(*I_j_*).
4. To estimate the boundaries of interval $J\approx \left[\min_{j}\;C_{j},\max_{j}\;C_{j}\right]$.
5. To estimate the marginal error of the value of function *f* caused by the inaccuracy of its arguments using the expression $\Delta C=\max_{j}\;\left|C_{j}-C_{0}\right|$.

When the Monte Carlo method is applied to study the distribution transformations, the appropriate value of the number *N* of iterations is about 10^6^ [35,48]. For the presented variant, *N* can be reduced to 10^3^ [49]. The confidence probability *P* for the interval *J* and corresponding value Δ*C* can be assessed by the following expression [49]:

$$P\approx {\left(\frac{\log2}{N}\right)}^{\frac{1}{N-1}}$$

.
